# A Weighted Linear Least Squares Location Method of an Acoustic Emission Source without Measuring Wave Velocity

**DOI:** 10.3390/s20113191

**Published:** 2020-06-04

**Authors:** Zilong Zhou, Yichao Rui, Xin Cai, Ruishan Cheng, Xueming Du, Jianyou Lu

**Affiliations:** 1 School of Resources and Safety Engineering, Central South University, Changsha 410083, China; zlzhou@csu.edu.cn (Z.Z.); ruiyichao@csu.edu.cn (Y.R.); chengruishan@csu.edu.cn (R.C.); jianyou.lu@csu.edu.cn (J.L.); 2 College of Water Conservancy and Environmental Engineering, Zhengzhou University, Zhengzhou 450001, China; dxm2019@zzu.edu.cn

**Keywords:** acoustic emission, source location, wave velocity, equation residuals

## Abstract

The location of an acoustic emission (AE) source is crucial for predicting and controlling potential hazards. In this paper, a novel weighted linear least squares location method for AE sources without measuring wave velocity is proposed. First, the governing equations of each sensor are established according to the sensor coordinates and arrival times. Second, a mean reference equation is established by taking the mean of the squared governing equations. Third, the system of linear equations can be obtained based on the mean reference equation, and their residuals are estimated to obtain their weights. Finally, the AE source coordinate is obtained by weighting the linear equations and inserting the parameter constraint. The AE location method is verified by a pencil lead break experiment, and the results show that the locating accuracy of the proposed method is significantly higher than that of traditional methods. Furthermore, the simulation test proves that the proposed method also has a better performance (location accuracy and stability) than the traditional methods under any given scale of arrival errors.

## 1. Introduction

Acoustic emission (AE) location technology is an important nondestructive testing method that is widely used in many applications, including underground tunnels, deep mining, environmental acoustics, and the petrochemical and aerospace industries [1,2,3,4,5,6,7,8,9]. Accurately identifying the location of microscopic fractures and damage is the scientific basis for researching the failure mechanism of materials, predicting rock bursts, and safely operating large industrial equipment [10,11,12,13,14,15,16,17]. Therefore, it is highly worthwhile to develop a high-precision AE source location method [18,19,20,21,22,23].

To date, numerous methods have been proposed to locate AE sources, including the maximum likelihood method [24], Inglada method [25], and spherical interpolation method [26]. These traditional approaches can achieve a good localization of AE sources with an accurate average wave velocity measured beforehand [27]. However, it is very difficult to determine the average wave velocity precisely due to the following three factors [28,29,30]. First, with the advance in engineering or progress in material stress testing, microcracks in the propagation medium continue to initiate and propagate, resulting in a real-time change in the average wave velocity. However, the real-time measurement of the average wave velocity is of great difficulty or even impossible in most scenarios, such as rock mechanics testing and micro-seismic monitoring. Second, the propagation medium is often heterogeneous and anisotropic, so the wave velocity is different in different directions. Moreover, it is hardly possible to measure the wave velocities of all paths in different directions. Therefore, the premeasured velocity of a specific path will not be equal to the average wave velocity of all paths. Third, the propagation path of the AE signal traveling from the source to the sensor will change with the location of the AE source. Therefore, the average wave velocity of these paths also varies by source location. Due to the influence of the above-mentioned three factors, the location methods with the pre-measured velocity will inevitably produce errors. Thus, there is an urgent need to eliminate the dependence on wave velocity measurements to improve the accuracy of AE source location.

In recent years, considerable efforts have been made to develop AE location methods without measuring wave velocity [31,32]. Das et al. [33] proposed a positioning framework for unknown wave velocity systems which located the AE source by updating the spatial position and the average wave velocity separately. In this method, only the coordinates of the AE sensors and the arrival times need to be determined, and the wave velocity of the material is not needed. Dong et al. [34] proposed a new location method using the arrival times of both P-waves and S-waves. Nonlinear equations of time difference were constructed first, and then the source and the average wave velocity were jointly inversed through multiple iterations. Dong et al. [35] further proposed a multistep location method for spatial sources without measuring the average wave velocity. This method could achieve a more stable location result after finding the optimal interval of the minimum and maximum velocities obtained from the masses of prior localizations. All these methods mentioned above can avoid the influence of velocity error and improve the location accuracy to some extent. However, the cost functions of all these methods are nonlinear and the iterative optimization algorithms must be used to search the optimal solution, including the geiger algorithm [36,37], simplex algorithm [38], and differential evolution algorithm [39]. The nature of iterative methods brings two major disadvantages [40,41]: first, the real-time application of these methods is poor due to the multiple iterations and calculations; second, these methods require a good initial guess to avoid the local convergence problem, but the selection of such an initial guess is difficult in actual engineering practice. Although some techniques, such as a gird search or Taylor second-order expansion, can be combined to improve the convergence, they will also incur more computing costs. To overcome these issues, Kundu et al. [42] proposed a closed-form method without measuring wave velocity based on sensor clusters, in which every three sensors were placed close to one another, forming a cluster at the vertices of an isosceles right triangle. Yin et al. [43] further improved this method by using “Z”-shaped sensor clusters, which could achieve a similar location accuracy with fewer sensors. These location methods were efficient to some extent, since the solving process was not affected by iterative algorithms. However, these methods still have the following limitations. First, these methods provide far less redundant constraints and are highly sensitive to arrival errors, so that a small error in arrival time will lead to a large location deviation. Second, due to the basic assumption that the inclination angles from the AE source to the sensors in the cluster are approximately same, the distance between the sensors in the cluster should be much smaller than the distance between the AE source and the sensor cluster. However, since the distance between the sensor cluster and the source is unknown, it is difficult to determine whether the distance meets the basic assumption. If these sensor clusters that do not meet, the basic assumption are used in this method, the positioning results will be rather poor. Third, the difference in arrival times for the sensors in the cluster are fairly small. It is difficult to accurately determine such small differences in noisy engineering practices. Therefore, the calculation of the inclination angle and the wave velocities will always fail, which will lead to a large deviation in the final location result. Finally, placing sensors in such a special array is very time and labor consuming or even impossible in many scenarios, such as micro-seismic positioning and rock mechanics tests. The above four problems severely limit the application of these methods. However, the following methods based on the average wave velocity model can efficiently avoid the above problems. For example, Dong et al. [44] derived the analytical solution of AE source location for a cuboid monitoring system without measuring the average wave velocity. Mahajan et al. [45] used six sensors to solve the analytical solution of the AE source for a more random sensor array. These methods are efficient and can achieve a better location result. Nevertheless, they are limited by the number of the AE sensors and cannot make full use of the extra sensors in random multi-sensor networks. To this end, Dong et al. [46,47] proposed comprehensive analytical solutions (CAS) to further improve the location accuracy. In this method, the preliminary location results of every six sensors are obtained first and then fitted by a logistic function. The coordinates corresponding to the extreme value of the logistic function are deemed the final location result. However, the logistic distribution assumption of the AE source coordinates are not optimal, which always leads to a large error in the final location result [18,48]. The non-iterative location method with unknown velocity (NIUV), by exploiting the least square principle, can further improve the location accuracy. However, due to the existence of multiple solutions, a priori knowledge is needed to determine the true solution [49,50]. Moreover, these above-mentioned methods ignore the estimation of the equation residuals caused by the arrival errors, resulting in a poor location result. The selection of the reference sensor also have a large influence on the location accuracy, and a small arrival error in the reference sensor can lead to a large deviation in the final location result [51].

To further improve the location accuracy, a weighted linear least squares solution of AE source location without measuring wave velocity is proposed. In this method, the mean reference equation is first established to linearize the governing equations. Then, the residuals are estimated to weigh these linear equations. Finally, the location result considering the parameter constraint is obtained by introducing an orthogonal projection matrix. The pencil lead break experiment and simulation analysis are combined to verify its effectiveness and accuracy.

## 2. Methods

**Figure 1:**
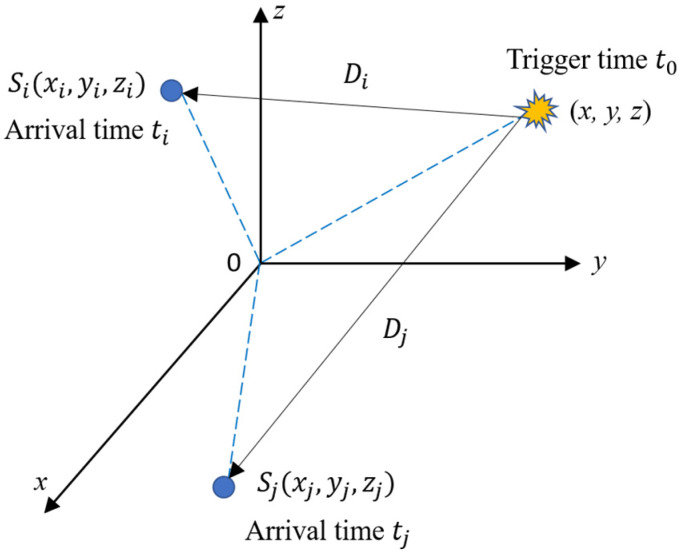
Spatial diagram illustrating the variables defined in the proposed method.

The method addressed here determines the location of an AE source with sensor coordinates and arrival times. Let $S_{i}\;\left(x_{i},\;y_{i},\;z_{i}\right)\;\left(i=1,\;2,\;\cdots ,M\right)$ denote the AE sensors in a location system and (x, y, z) represent the AE source to be determined (see Figure 1). If the noise-free value of {·} is denoted as ${\left\{\cdot \right\}}^{o}$, the arrival measurement $t_{i}$ of the sensor $S_{i}$ can be modeled as:

(1) $$t_{i}=t_{i}^{o}+n_{i},\;\;i=1,\;2,\cdots ,M,$$

where the measurement error $n_{i}$ is assumed to be a zero-mean Gaussian process.

Our goal is to determine the AE source using these M arrival measurements. The governing equation of the AE sensor $S_{i}$ is denoted as:

(2) $$D_{i}=v\left(t_{i}-t_{0}\right),\;\;i=1,\;2,\cdots ,M,$$

where $D_{i}$ denotes the distance between the AE source and the AE sensor, $S_{i}$, and its expression is $D_{i}=\sqrt{{\left(x_{i}-x\right)}^{2}+{\left(y_{i}-y\right)}^{2}+{\left(z_{i}-z\right)}^{2}}$. $t_{i}$ is the arrival time of the sensor $S_{i}$, $t_{0}$ is the trigger time of the AE source, and v is the average wave velocity.

To linearize the governing Equation (2), the traditional methods always choose one of the sensors as the reference sensor. However, the selection of the reference sensor always leads to a biased location result [51]. To reduce the influence of reference sensor on the final location results, the governing Equation (2) of all the sensors are squared and added up. Then, the result is divided by the number *M* of these equations to obtain the average reference equation:

(3) $$\frac{1}{M}\sum_{j=1}^{M}\left[{\left(x_{j}-x\right)}^{2}+{\left(y_{j}-y\right)}^{2}+{\left(z_{j}-z\right)}^{2}\right]=v^{2}\frac{1}{M}\sum_{j=1}^{M}{\left(t_{j}-t_{0}\right)}^{2}.$$

Subtracting Equation (3) from squared Equation (2) to obtain *M* linear equations:

(4) $$L_{i}=a_{i}x+b_{i}y+c_{i}z+d_{i}K+e_{i}V,$$

where:

$$L_{i}=x_{i}^{2}+y_{i}^{2}+z_{i}^{2}-\left(\frac{1}{M}\overset{M}{\underset{j=1}{\sum }}x_{j}^{2}+\frac{1}{M}\overset{M}{\underset{j=1}{\sum }}y_{j}^{2}+\frac{1}{M}\overset{M}{\underset{j=1}{\sum }}z_{j}^{2}\right)$$

$$a_{i}=2x_{i}-2\frac{1}{M}\overset{M}{\underset{j=1}{\sum }}x_{j}$$

$$b_{i}=2y_{i}-2\frac{1}{M}\overset{M}{\underset{j=1}{\sum }}y_{j}$$

$$c_{i}=2z_{i}-2\frac{1}{M}\overset{M}{\underset{j=1}{\sum }}z_{j}$$

$$d_{i}=-2{\left(t_{i}-\frac{1}{M}\overset{M}{\underset{j=1}{\sum }}t_{j}\right)}^{\;}$$

$$e_{i}=t_{i}^{2}-\frac{1}{M}\overset{M}{\underset{j=1}{\sum }}t_{j}^{2}$$

$$V=v^{2}$$

$$K=Vt_{0}$$

and

i=1, 2,⋯,M

In the presence of arrival noise, the equation residuals are added to Equation (4) and expressed in matrix form as:

(5) φ=L−BK−CV – Aθ,

where φ is the vector of the equation residuals, $A=2\left[\begin{array}{ccc} a_{1} & b_{1} & c_{1}\\ a_{2} & b_{2} & c_{2}\\ \vdots & \vdots & \vdots \\ a_{M} & b_{M} & c_{M} \end{array}\right]$, $B=2\left[\begin{array}{c} d_{1}\\ d_{2}\\ \vdots \\ d_{M} \end{array}\right]$, $C=\left[\begin{array}{c} e_{1}\\ e_{2}\\ \vdots \\ e_{M} \end{array}\right]$, $\theta =\left[\begin{array}{c} x\\ y\\ z \end{array}\right]$, and $L=\left[\begin{array}{c} L_{1}\\ L_{2}\\ \vdots \\ L_{M} \end{array}\right]$.

Without considering the constraint in Equation (3) and the residual estimation, the least square solutions of the intermediate variables *V* and *K*, denoted by $K^{\left(1\right)}$ and $V^{\left(1\right)}$, can be readily solved from Equation (5) by:

(6) $$\left[\begin{array}{c} K^{\left(1\right)}\\ V^{\left(1\right)} \end{array}\right]={\left(D^{T}{P^{\prime }}_{A}^{⊥}D\right)}^{-1}D^{T}{P^{\prime }}_{A}^{⊥}L,$$

where  D=[B,C] and ${P^{\prime }}_{A}^{⊥}=I-A{\left(A^{T}A\right)}^{-1}A^{T}$; ${P^{\prime }}_{A}^{⊥}$ is the idempotent projection matrix $\left({{P^{\prime }}_{A}^{⊥}}^{2}={P^{\prime }}_{A}^{⊥}\right)$, which removes components in the space spanned by the columns of *A*.

The least square solution of AE source coordinate θ**,** denoted by $\theta^{\left(1\right)}$, can also be given by:

(7) $$\theta^{\left(1\right)}={\left(A^{T}{P^{\prime }}_{D}^{⊥}A\right)}^{-1}A^{T}{P^{\prime }}_{D}^{⊥}L,\;$$

where ${P^{\prime }}_{D}^{⊥}=I-D{\left(D^{T}D\right)}^{-1}D^{T}$ and ${P^{\prime }}_{D}^{⊥}$ is the idempotent projection matrix $\left({{P^{\prime }}_{D}^{⊥}}^{2}={P^{\prime }}_{D}^{⊥}\right)$, removing components in the space spanned by the columns of *D*. However, the ordinary least solution in Equation (7) is not optimal; the estimation of the equation residuals and the constraint among parameters *x*, *y*, *z*, *V*, and *K* will be further exploited to improve the location accuracy.

When Equation (1) is used to express $t_{i}$ as $t_{i}^{o}+n_{i}$ and the quadratic term is ignored, φ in Equation (5) is found to be:

(8) $$\varphi \approx 2V^{o}\left(t_{i}^{o}-t_{0}\right)n_{i}-\frac{1}{M}\overset{M}{\underset{j=1}{\sum }}\left[2V^{o}\left(t_{j}^{o}-t_{0}\right)n_{j}\right].$$

The first term is the Gaussian random vector and its mean value (i.e., the second term) is a constant [41]. Therefore, the equation residual φ is still the Gaussian random vector, with an approximate covariance matrix of:

(9) $$\Psi =E\left(\varphi \varphi^{T}\right)=4{V^{o}}^{2}PNP,$$

where ${\left\{\cdot \right\}}^{T}$ expresses the transpose of {·}, $P=\mathrm{diag}\left(t_{1}^{o}-t_{0},t_{2}^{o}-t_{0},\cdots ,t_{M}^{o}-t_{0}\right)$, and N is the covariance matrix of $n_{i},$ which can be determined using the power spectra. For simplicity, the source signal and noises for the arrival times are assumed to be white processes, and the signal-to-noise ratios at all $n_{i}$ are identical, thus:

(10) $$N=E\left(nn^{T}\right)=\left[\begin{array}{ccc} 1 & \cdots & 0\\ \vdots & \ddots & \vdots \\ 0 & \cdots & 1 \end{array}\right].$$

However, the parameter Ψ in Equation (9) remains to be determined due to the unknown $t_{i}^{o}-t_{0}$ and $V^{o}$. Then, the result $V^{\left(1\right)}$ in Equation (6) is used to approximate the parameter $V^{o}$, and the parameter $t_{i}^{o}-t_{0}$ is approximated by:

(11) $$t_{i}^{o}-t_{0}\approx \frac{\sqrt{{\left(\theta_{1}^{\left(1\right)}-x_{i}\right)}^{2}+{\left(\theta_{2}^{\left(1\right)}-y_{i}\right)}^{2}+{\left(\theta_{3}^{\left(1\right)}-z_{i}\right)}^{2}}}{V^{\left(1\right)}},$$

where $\theta_{1}^{\left(1\right)}$, $\theta_{2}^{\left(1\right)}$, and $\theta_{3}^{\left(1\right)}$ denotes the first, second, and third elements of $\theta^{\left(1\right)}$ in Equation (7).

After determining the covariance matrix Ψ, the weight matrix of Equation (5) can be obtained by:

(12) $$W=\Psi^{-1},$$

where ${\left\{\cdot \right\}}^{-1}$ denotes the inverse of matrix {·}. Then, the weighted least square solution of θ in terms of *V*, denoted by $\theta^{\left(2\right)}$, is given by:

(13) $$\theta^{\left(2\right)}={\left(A^{T}P_{B}^{⊥}WP_{B}^{⊥}A\right)}^{-1}A^{T}P_{B}^{⊥}WP_{B}^{⊥}\left(L-CV\right)=p-qV,$$

where $P_{B}^{⊥}=I-B{\left(B^{T}WB\right)}^{-1}B^{T}W$, and $P_{B}^{⊥}$ is the weighted orthogonal projection matrix, which removes components in the space spanned by the column of *B* in different weights; $p={\left[\begin{array}{c} \begin{array}{ccc} p_{1} & p_{2} & p_{3} \end{array} \end{array}\right]}^{T}$ and $q={\left[\begin{array}{c} \begin{array}{ccc} q_{1} & q_{2} & q_{3} \end{array} \end{array}\right]}^{T}$.

The weighted least square solution of the intermediate variable K in terms of V, denoted by $K^{\left(2\right)}$, is given by:

(14) $$K^{\left(2\right)}={\left(B^{T}P_{A}^{⊥}WP_{A}^{⊥}B\right)}^{-1}B^{T}P_{A}^{⊥}WP_{A}^{⊥}\left(L-CV\right)=p_{4}-q_{4}V,$$

where $P_{A}^{⊥}=I-A{\left(A^{T}WA\right)}^{-1}A^{T}W$, and $P_{A}^{⊥}$ is the weighted orthogonal projection matrix, removing components in the space spanned by the columns of *A* at different weights.

Substituting Equations (13) and (14) into the constraint of Equation (3), the cubic equation of variable *V* can be obtained:

(15) $$aV^{3}+bV^{2}+cV+d=0,$$

where

$$a=q_{1}^{2}+q_{2}^{2}+q_{3}^{2},$$

$$b=-q_{4}^{2}-2q_{4}\frac{1}{M}\overset{M}{\underset{j=1}{\sum }}t_{j}-\frac{1}{M}\overset{M}{\underset{j=1}{\sum }}t_{j}^{2}-2p_{1}q_{1}-2p_{2}q_{2}-2p_{3}q_{3}+2q_{1}\frac{1}{M}\overset{M}{\underset{j=1}{\sum }}x_{j}+2q_{2}\frac{1}{M}\overset{M}{\underset{j=1}{\sum }}y_{j}+2q_{3}\frac{1}{M}\overset{M}{\underset{j=1}{\sum }}z_{j},$$

$$c=p_{1}^{2}-2p_{1}\frac{1}{M}\overset{M}{\underset{j=1}{\sum }}x_{j}+p_{2}^{2}-2p_{2}\frac{1}{M}\overset{M}{\underset{j=1}{\sum }}y_{j}+p_{3}^{2}-2p_{3}\frac{1}{M}\overset{M}{\underset{j=1}{\sum }}z_{j}+\frac{1}{M}\overset{M}{\underset{j=1}{\sum }}x_{j}^{2}+\frac{1}{M}\overset{M}{\underset{j=1}{\sum }}y_{j}^{2}+\frac{1}{M}\overset{M}{\underset{j=1}{\sum }}z_{j}^{2}+2p_{4}q_{4}+2p_{4}\frac{1}{M}\overset{M}{\underset{j=1}{\sum }}t_{j},$$

and

$$\;d=-p_{4}^{2}.\;$$

By solving the cubic equations, the variable *V* in closed form, denoted by $V^{\left(2\right)}$, can be readily solved. There are three solutions which are then substituted into Equation (13) to obtain the AE source coordinates $\theta^{\left(2\right)}$. However, only one real numerical solution can be determined as the final location result. When there is more than one real numerical solution, the one closest to the least square solution $\theta^{\left(1\right)}$ is retained.

Moreover, the optimal average wave velocity *v* can be further obtained by:

(16) $$v=\sqrt{V^{\left(2\right)}}.$$

**Figure 2:**
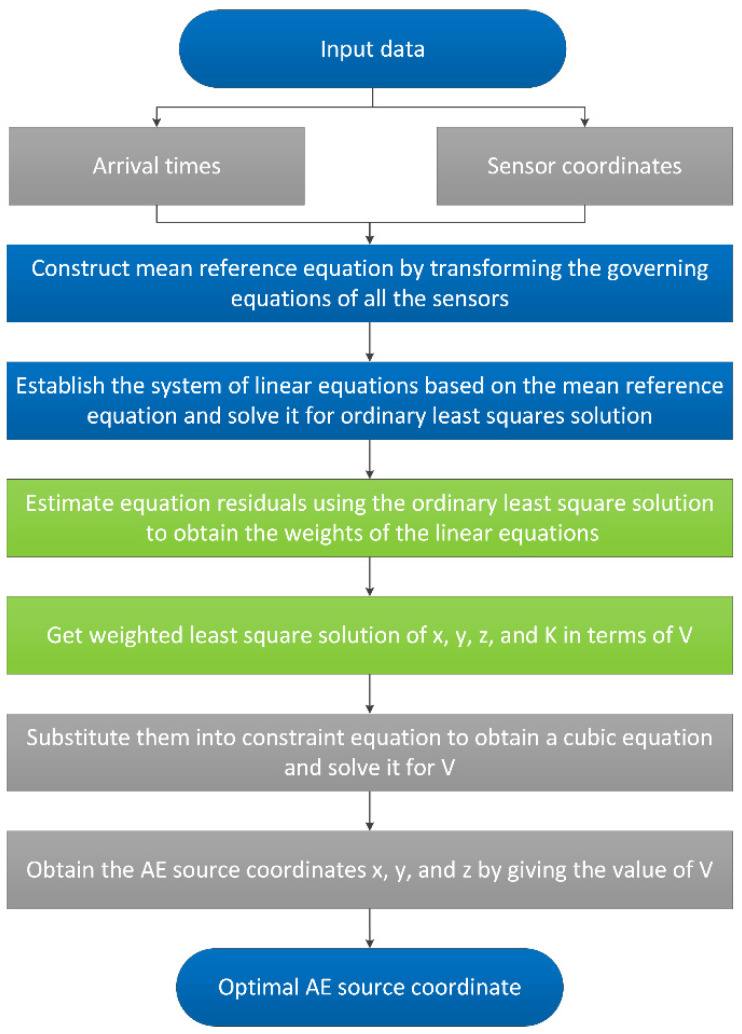
The whole location process of the proposed method.

The whole procedure of the new method is shown in Figure 2.

## 3. Experimental Verification by Pencil Lead Breaks

**Figure 3:**
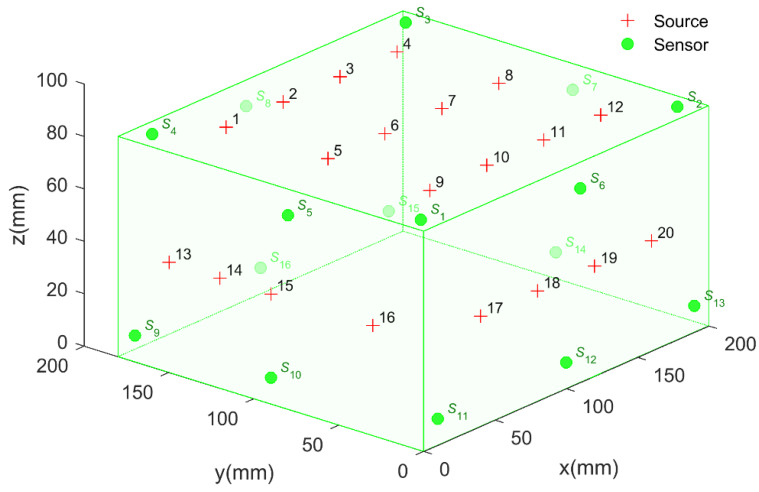
The layout of the AE sensors and sources in the pencil lead break experiment.

**Figure 4:**
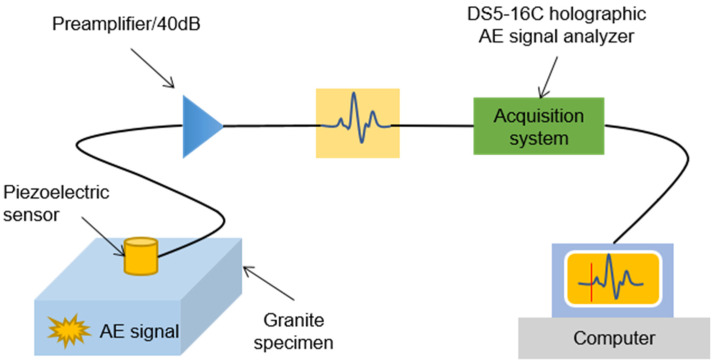
Acquisition process of the AE signal generated by pencil lead breaks.

A pencil lead break experiment was performed on a monitoring system with a 200 × 179 × 84 mm granite block to verify the feasibility of the proposed method, as shown in Figure 3. Sixteen sensors 2343 mounted on the monitoring system, and their coordinates are shown in Table 1. Moreover, 20 AE sources were generated on the block surface by pencil lead breaks, and their coordinates can be seen in Table 2. It should be noted that a 0.5 mm hard-black pencil lead was used, and the lead-breaking angle with respect to the granite block surface was 30°. Then, the acoustic waves were received by the piezoelectric sensor, amplified by a gain of 40 dB, and collected by a DS5-16C Holographic AE Signal Analyzer. Finally, the acoustic waves were stored in a computer for further analysis. The entire acquisition process of acoustic waves is shown in Figure 4.

**Figure 5:**
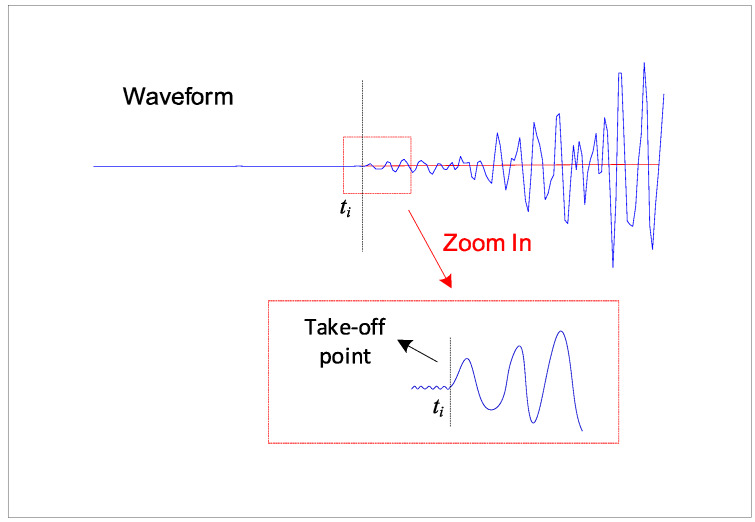
The principle of the selection of arrival times.

Before locating an AE source, the arrival times should be determined first. Because the power of the P wave is far higher than that of environmental noise, there will be a significant take-off at the corresponding waveform with the P wave arriving. The corresponding time of the first take-off point is generally determined as the time of arrival. Figure 5 shows the principle of the selection of arrival times.

**Figure 6:**
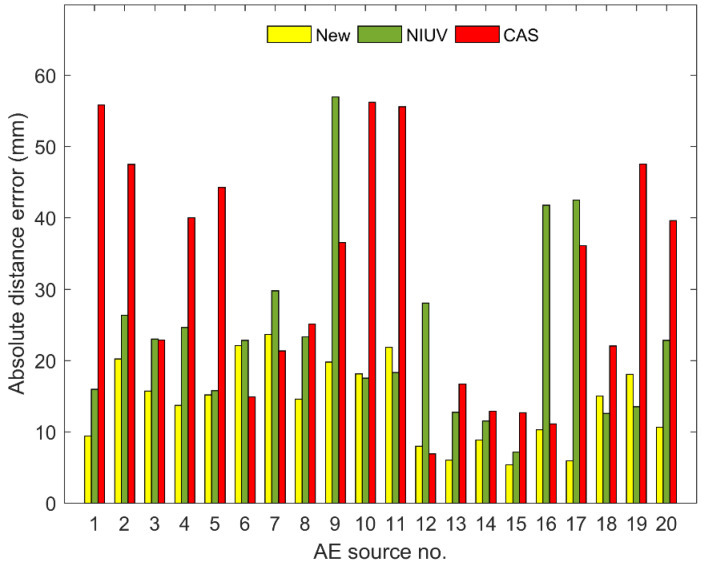
The comparison of location errors for AE source nos. 1 to 20 as determined by the new method and two traditional methods.

**Figure 7:**
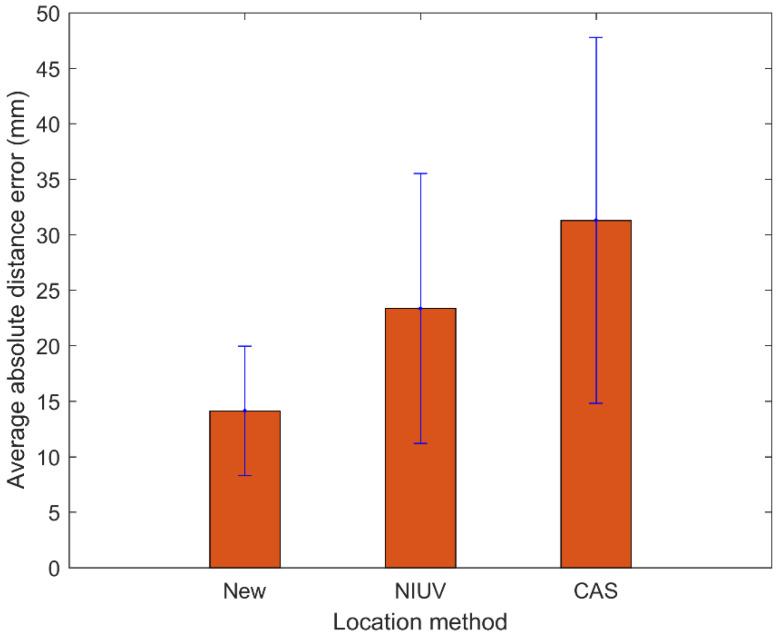
Average absolute distance errors and standard deviations for the location results of 20 AE sources determined by three methods.

Figure 6 compares the location errors for AE sources nos. 1 to 20 as determined by the new method and two traditional methods. It can be seen that the absolute distance errors of the proposed method are generally smaller than those of the traditional methods, except for the AE sources 10, 11, 18, and 19 for the NIUV method and AE sources 6, 7, and 12 for the CAS method. Moreover, the maximum location errors of the CAS and NIUV methods are both more than 50 mm, while that of the new method is less than 25 mm. Therefore, the location performance of the new method is far better than that of the traditional methods. Figure 7 shows the average absolute distance errors and standard deviations for the location results of 20 AE sources determined by the three methods. It can be seen that the average absolute distance error and standard deviation of the proposed method are both smaller than those of the traditional methods, which further demonstrates the higher location accuracy and stability of the proposed method compared with the traditional methods. The detailed AE source coordinates of the location results for the three methods are shown in Table 2.

## 4. Simulation Analysis

The arrival errors significantly affected the location accuracy [32,52]. Due to the uncontrollability of arrival errors, a quantitative analysis of the influence of arrival errors on the location accuracy cannot be performed through the pencil lead break experiment. Herein, a simulation approach is adopted to evaluate the locating performance of the proposed method.

**Figure 8:**
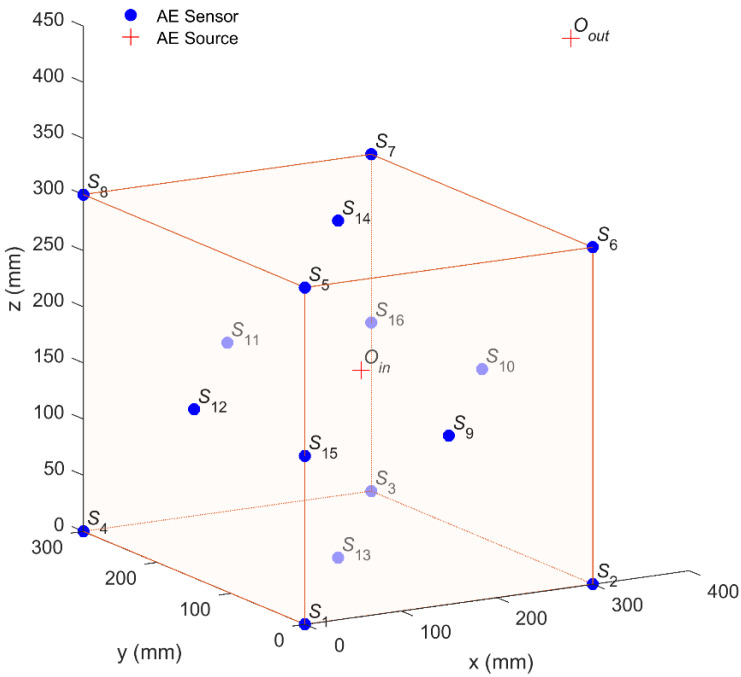
The sensor array and two AE sources inside and outside the array, respectively.

As shown in Figure 8, a cubic monitoring system with a side length of 300 mm containing 16 AE sensors is established. The coordinates of the sensors are shown in Table 3. Moreover, two virtual AE sources were used for this simulation: one was set inside the sensor array with the coordinate of $O_{in}$ (120, 80, 190 mm), and another was set outside the sensor array with the coordinate of $O_{out}$ (400, 160, 430 mm). The arrivals with the error scales (i.e., error standard deviations) of 0.3, 0.6, 0.9, 1.2, and 1.5 μs were designed as noise disturbances. Each AE source location was repeated 100 times by changing the random errors in arrivals to obtain reliable statistical conclusions.

**Figure 9:**
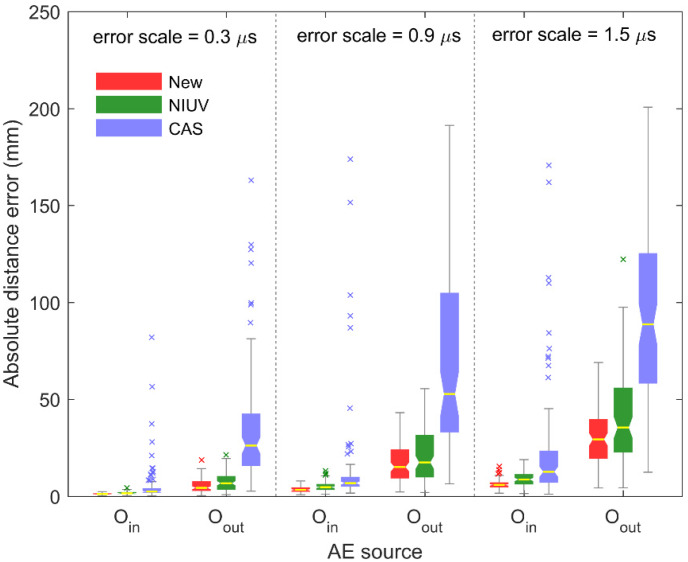
The comparison of the average absolute distance errors between the new method and two traditional methods using arrivals with error scales of 0.3, 0.9, and 1.5 μs.

Figure 9 shows the comparison of absolute distance errors of the proposed method and the traditional methods using arrivals with error scales of 0.3, 0.9, and 1.5 μs. For each box, the position of the notch is the median; the box extends vertically between the lower quartile and upper quartile, the whiskers extend to the most extreme data that are not considered abnormal results, and the abnormal results denoted by the cross markers “×” are plotted individually. In this figure, the lower the position of the notch, the higher the location accuracy, and the shorter the box, the more stable the location accuracy. In can be seen that, regardless of whether the AE source is in or out of the array, the box of the new method is always at a lower position and shorter than the NIUV and CAS methods under different error scales. It indicates that the new method always has more accurate and stable location results under different error scales than the NIUV and CAS methods whether the AE source is inside or outside the sensor array. Because the new method considers the influence of the reference sensor, estimates the equation residuals, and embeds the parameter constraint, better positioning results are obtained.

**Figure 10:**
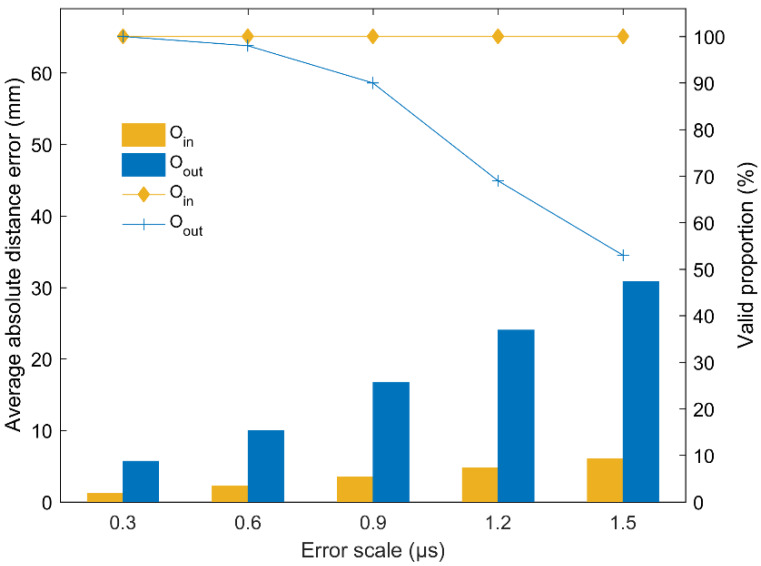
The average absolute distance errors and valid location proportions of the proposed method for the AE sources inside and outside the array under different error scales.

Figure 10 shows the average absolute distance errors and valid proportions of the proposed method under different error scales. The valid proportion is the proportion of the location results whose absolute distance errors are less than 10% of the side length of the monitoring area. From this figure, it can be seen that the average absolute distance errors of the AE source inside the array are smaller and increase more slowly with the increase in the error scales than those of the AE source outside the array. In addition, the location results of the AE source inside the array are more stable and all of them have 100% valid proportions. With the increase in error scales, the valid proportions rapidly decrease to close to 50% for the AE source outside the array. The fundamental reason behind this observation is that the non-uniform characteristic of the hyperboloid field associated with AE source location leads to the non-uniform amplifying effect of the sensor array on the arrival errors. Specifically, due to the existence of arrival errors, the calculated AE source falsely falls on the adjacent hyperboloids, resulting in positioning errors. Since the hyperboloid density inside the sensor array is much higher than that outside the sensor array (i.e., two adjacent hyperboloids inside the sensor array are closer together) and the location error inside the sensor array is generally smaller than that outside the sensor array [53], it is therefore suggested that the sensor array should be arranged to surround the monitored area as much as possible, especially in practical engineering.

## 5. Conclusions

In this paper, a weighted linear least squares location method for an AE source without measuring wave velocity is proposed. First, the mean reference equation is established by transforming the governing equations for all the sensors, which are used to obtain the system of the linear equations. Second, the ordinary least squares solutions are obtained by solving this linear system and are used to estimate the equation residuals. Third, the equation residuals are transformed into weights to obtain the weighted least squares solutions in terms of velocity squared. Finally, the optimal positioning result is obtained by combining the weighted least square solution with the parameter constraint. The proposed method highlights the following advantages: (1) the influence of the measurement error of the average wave velocity on the location accuracy is eliminated; (2) the equation residuals are estimated to obtain the weights, and a better location result is obtained; (3) the selection of the reference sensor is avoided, and the biasness caused by the reference sensor is reduced; (4) the AE source coordinate and the intermediate variables are separately solved by orthogonal projection, lowering their mutual influence; (5) the closed-form solution of the AE source coordinate is obtained, avoiding the compute-intensive work and nonconvergence. The pencil lead break experiment verifies that a more stable and accurate location result can be gained by the proposed method compared with the traditional methods. The simulation results indicate that the new method always has a better location performance than the traditional methods under different error scales, whether the AE source is inside the sensor array or not. Actually, with the increase in the error scales, the average absolute distance errors of the inside AE source are always smaller and increase more slowly than those of the outside AE source. Herein, the sensor array is suggested to surround the monitored area as much as possible.

The proposed method is not limited by the type of transmitted wave. As long as the arrival time of the transmitted wave and the coordinates of the sensor are determined, the location of the source can be obtained. However, there is still a limitation because the basic assumption of this method is that the propagation path of the AE signal from the source to the sensor is a straight line, while the actual propagation path is commonly complicated. Therefore, future research should be carried out to address this limitation.

## Figures and Tables

**Table 1:** The coordinates of 16 acoustic emission (AE) sensors used in the experiment of pencil lead breaks.

Sensor No.	*x* (mm)	*y* (mm)	*z* (mm)
1	10	10	84
2	190	10	84
3	190	170	84
4	12	170	84
5	0	80	74
6	110	0	74
7	200	80	74
8	90	180	74
9	0	170	10
10	0	90	10
11	10	0	10
12	100	0	10
13	190	0	10
14	200	90	10
15	190	180	10
16	100	180	10

**Table 2:** Location results of AE sources nos. 1 to 20 solved by three methods.

AE Source No.	Methods	True Source Coordinates				AE Source No.	Methods	True Source Coordinates			
		*x* (mm)	*y* (mm)	*z* (mm)	Absolute Distance Error (mm)			*x* (mm)	*y* (mm)	*z* (mm)	Absolute Distance Error (mm)
1	True position	40.00	150.00	84.00	**-**	11	True position	120.00	30.00	84.00	-
	New	39.41	155.99	91.20	9.39		New	122.41	27.06	105.49	21.83
	NIUV	45.06	153.36	98.76	15.96		NIUV	121.12	28.89	102.25	18.32
	CAS	22.93	184.95	124.06	55.84		CAS	124.99	−7.32	124.90	55.60
2	True position	80.00	150.00	84.00	-	12	True position	160.00	30.00	84.00	-
	New	81.71	157.52	102.72	20.25		New	165.02	26.15	88.87	7.98
	NIUV	81.63	154.09	109.97	26.34		NIUV	168.22	19.31	108.64	28.09
	CAS	75.08	176.28	123.30	47.53		CAS	162.45	27.51	89.94	6.90
3	True position	120.00	150.00	84.00	-	13	True position	0.00	150.00	42.00	-
	New	116.21	139.77	95.29	15.70		New	4.38	153.93	43.16	6.00
	NIUV	113.24	137.39	102.04	23.02		NIUV	−3.45	161.10	47.16	12.72
	CAS	109.53	137.50	100.06	22.89		CAS	−1.24	166.26	45.55	16.69
4	True position	160.00	150.00	84.00	-	14	True position	0.00	120.00	42.00	-
	New	162.47	155.20	96.46	13.72		New	8.07	119.25	45.63	8.88
	NIUV	157.51	149.70	108.51	24.64		NIUV	5.99	120.77	51.78	11.49
	CAS	170.47	167.40	118.50	40.03		CAS	−5.73	122.43	53.30	12.90
5	True position	40.00	90.00	84.00	-	15	True position	0.00	90.00	42.00	-
	New	47.66	91.18	97.05	15.18		New	−5.03	89.00	43.61	5.37
	NIUV	52.98	92.08	92.76	15.79		NIUV	3.59	88.76	48.05	7.14
	CAS	−1.69	75.60	87.68	44.27		CAS	−1.31	87.30	54.35	12.71
6	True position	80.00	90.00	84.00	-	16	True position	0.00	30.00	42.00	-
	New	82.90	92.47	105.77	22.10		New	−2.96	20.74	45.29	10.26
	NIUV	82.57	93.42	106.44	22.84		NIUV	−29.37	0.31	43.72	41.80
	CAS	91.28	98.21	89.18	14.88		CAS	−2.17	19.16	41.00	11.10
7	True position	120.00	90.00	84.00	-	17	True position	40.00	0.00	42.00	-
	New	120.37	92.86	107.47	23.65		New	40.78	−4.08	46.24	5.94
	NIUV	120.96	92.67	113.64	29.78		NIUV	64.39	33.30	52.26	42.53
	CAS	120.35	93.10	105.14	21.37		CAS	19.41	−29.33	46.59	36.12
8	True position	160.00	90.00	84.00	-	18	True position	80.00	0.00	42.00	-
	New	159.08	89.84	98.57	14.60		New	81.59	−14.73	44.64	15.04
	NIUV	155.95	85.77	106.57	23.32		NIUV	85.10	8.22	50.02	12.57
	CAS	149.81	72.34	98.68	25.13		CAS	89.04	−19.24	47.82	22.04
9	True position	40.00	30.00	84.00	-	19	True position	120.00	0.00	42.00	-
	New	32.69	18.83	98.59	19.78		New	117.06	−17.83	41.36	18.08
	NIUV	10.69	−4.62	118.43	56.95		NIUV	110.03	8.16	37.85	13.54
	CAS	26.84	13.36	113.73	36.52		CAS	111.05	−30.89	6.96	47.56
10	True position	80.00	30.00	84.00	-	20	True position	160.00	0.00	42.00	-
	New	79.71	29.91	102.10	18.10		New	156.77	−9.53	45.37	10.61
	NIUV	83.60	33.97	100.68	17.52		NIUV	156.78	−21.83	47.76	22.80
	CAS	76.27	8.47	135.82	56.24		CAS	167.59	−38.22	34.72	39.64

**Table 3:** The coordinates of 16 AE sensors in the simulation test.

Sensor No.	*x* (mm)	*y* (mm)	*z* (mm)
1	0	0	0
2	300	0	0
3	300	300	0
4	0	300	0
5	0	0	300
6	300	0	300
7	300	300	300
8	0	300	300
9	150	0	150
10	300	150	150
11	150	300	150
12	0	150	150
13	150	150	0
14	150	150	300
15	0	0	150
16	300	300	150
